# Polyaxial stress-dependent permeability of a three-dimensional fractured rock layer

**DOI:** 10.1007/s10040-017-1624-y

**Published:** 2017-07-05

**Authors:** Qinghua Lei, Xiaoguang Wang, Jiansheng Xiang, John-Paul Latham

**Affiliations:** Department of Earth Science and Engineering, Imperial College London, London, UK

**Keywords:** Fractured rocks, Stress, FEMDEM, Hydraulic properties, Heterogeneity

## Abstract

A study about the influence of polyaxial (true-triaxial) stresses on the permeability of a three-dimensional (3D) fractured rock layer is presented. The 3D fracture system is constructed by extruding a two-dimensional (2D) outcrop pattern of a limestone bed that exhibits a ladder structure consisting of a “through-going” joint set abutted by later-stage short fractures. Geomechanical behaviour of the 3D fractured rock in response to in-situ stresses is modelled by the finite-discrete element method, which can capture the deformation of matrix blocks, variation of stress fields, reactivation of pre-existing rough fractures and propagation of new cracks. A series of numerical simulations is designed to load the fractured rock using various polyaxial in-situ stresses and the stress-dependent flow properties are further calculated. The fractured layer tends to exhibit stronger flow localisation and higher equivalent permeability as the far-field stress ratio is increased and the stress field is rotated such that fractures are preferentially oriented for shearing. The shear dilation of pre-existing fractures has dominant effects on flow localisation in the system, while the propagation of new fractures has minor impacts. The role of the overburden stress suggests that the conventional 2D analysis that neglects the effect of the out-of-plane stress (perpendicular to the bedding interface) may provide indicative approximations but not fully capture the polyaxial stress-dependent fracture network behaviour. The results of this study have important implications for understanding the heterogeneous flow of geological fluids (e.g. groundwater, petroleum) in subsurface and upscaling permeability for large-scale assessments.

## Résumé

Une étude sur l’influence des tensions polyaxiales (triaxiales véritables) sur la perméabilité d’une couche de roche fracturée en 3D est. présentée. Le système de fractures 3D est. construit en extrayant le motif d’affleurement 2D d’un niveau calcaire qui présente une structure en échelle consistant en un ensemble de joints parallèles contigu à des fractures courtes d’un stade ultérieur. Le comportement géomécanique de la roche fracturée en 3D en réponse aux contraintes in-situ est. modélisé par la méthode des éléments finis discrets, qui peut saisir la déformation des blocs de matrice, les variations du champ de contraintes, la réactivation de fractures préexistantes rugueuses et la propagation de nouvelles fissures. Une série de simulations numériques est. conçue pour imposer des contraintes à une roche fracturée en utilisant différentes tensions in situ polyaxiales et les propriétés d’écoulement dépendantes des tensions sont ensuite calculées. La couche fracturée a tendance à présenter une localisation plus forte des flux et une perméabilité équivalente supérieure lorsque le rapport des contraintes du champ éloigné augmente et que le champ de contraintes pivote de telle manière que les fractures sont orientées préférentiellement en faveur du cisaillement. La dilatation par cisaillement de fractures préexistantes a des effets dominants sur la localisation du flux dans le système, tandis que la propagation de nouvelles fractures a des impacts mineurs. Le rôle de la contrainte liée à la surcharge suggère que l’analyse conventionnelle 2D qui néglige l’effet de la contrainte hors plan (perpendiculaire à l’interface de stratification) peut fournir des approximations indicatives mais ne traduit pas entièrement le comportement du réseau de fractures soumis à des tensions polyaxiales. Les résultats de cette étude ont d’importantes implications pour comprendre l’écoulement hétérogène de fluides géologiques (par ex. eau souterraine, pétrole) en milieu souterrain et pour améliorer la perméabilité pour des évaluations à grande échelle.

## Resumen

Se presenta un estudio sobre la influencia de los esfuerzos poliaxiales (triaxiales verdaderas) sobre la permeabilidad de una capa tridimensional (3D) de rocas fracturadas. El sistema de fractura en 3D se construye mediante la extrusión de un patrón de afloramiento 2D de un lecho de caliza que exhibe una estructura de escalera que consiste en un conjunto articulado limitado por fracturas cortas de una etapa posterior. El comportamiento geomecánico de la roca fracturada en 3D en respuesta a los esfuerzos in situ es modelado por el método de elementos finitos y discretos, que puede capturar la deformación de bloques de matriz, la variación de campos de esfuerzos, la reactivación de fracturas irregulares preexistentes y la propagación de nuevas grietas. Una serie de simulaciones numéricas está diseñada para cargar la roca fracturada utilizando diferentes esfuerzos poliaxiales in situ y se calculan además las propiedades de flujo dependientes del esfuerzo. La capa fracturada tiende a exhibir una localización de flujo más fuerte y una permeabilidad equivalente más alta a medida que se incrementa la relación del esfuerzo en el campo lejano y se hace girar el campo de esfuerzo de tal manera que las fracturas se orientan preferentemente para el cizallamiento. La dilatación por cizalladura de fracturas preexistentes tiene efectos dominantes sobre la localización del flujo en el sistema, mientras que la propagación de nuevas fracturas tiene impactos menores. El papel del esfuerzo de la sobrecarga sugiere que el análisis 2D convencional que descuida el efecto del esfuerzo fuera del plano (perpendicular a la interfase del lecho) puede proporcionar aproximaciones indicativas pero no capturar completamente el comportamiento de la red de fractura dependiente del esfuerzo poliaxial. Los resultados de este estudio tienen importantes implicancias para la comprensión del flujo heterogéneo de fluidos geológicos (por ejemplo, aguas subterráneas, petróleo) en el subsuelo y en el escalado de la permeabilidad para evaluaciones a gran escala.

## 摘要

本文论述了多轴(真三轴)应力对三维裂隙岩层渗透率影响的研究成果。该石灰岩层具有阶梯结构,早期裂缝贯穿整个系统,后期较短的裂缝毗连早期裂缝。通过对两维露头 的几何沿岩层方向进行延申,建立了三维裂隙系统的几何模型。通过有限-离散元方法模拟了现场应力作用下三维裂隙岩体的力学特性,该方法能捕获岩块变形、应力场变化、自然裂缝的变形以及新裂隙的扩展。本文 设计了一系列的数值模拟实验,模拟加载多种多轴现场应力,进一步分析了渗透率对应力场的依赖性。随着远场应力比的 增加以及应力场的旋转,有些裂缝表现出很强的滑移,岩层也因此表现出很强的局部流动及很高的渗透率。研究表明,已有裂缝的滑移及剪涨效应对渗透率有非常重要的影响,而新裂缝的扩展影响较小。本文中发现的纵向应力效应表明,忽略平面外应力(垂直于层理界面)影响的传统二维分析可能提供了象征性的近似值,但无法完全捕获取决于多轴应力的裂缝网络的特性。本研究的结果对于了解地质流体(如地下水、石油)的不均匀流动、预测大尺度渗透率等具有重要的意义。

## Resumo

Um estudo é apresentado sobre a influência de esforço poliaxial (verdadeiramente triaxial) sobre a permeabilidade de uma camada de rocha fraturada tridimensional. O sistema fraturado 3D é construído extraindo-se um padrão de afloramento 2D de uma camada de calcário que exibe uma estrutura de escada consistindo de um conjunto de juntas interceptadas posteriormente por fraturas pequenas. O comportamento geomecânico 3D da rocha fraturada em resposta aos esforços locais é modelado pelo método de elementos discretos finitos, que pode capturar a deformação da matriz dos blocos, variação do campo de esforços, reativação de fraturas pré-existentes e propagação de novas rachaduras. Uma serie de simulações numéricas é desenvolvida para sobrecarregar a rocha fraturada usando diversos esforços poliaxiais locais e as propriedades de fluxo dependente dos esforços são posteriormente calculadas. A camada fraturada tende a mostrar forte fluxo localizado e maior permeabilidade equivalente assim que a taxa de esforço do campo externo é aumentada e o campo de esforço é rotacionado de forma que as fraturas são preferencialmente orientadas para cisalhamento. A dilatação por cisalhamento de fraturas pré-existentes tem efeitos dominantes no sistema de fluxo localizado, enquanto que a propagação de novas fraturas tem um impacto menor. O efeito do esforço do sobrecarregamento sugere que a análise 2D convencional que negligencia os efeitos dos esforços de fora do plano (perpendicular à interface do acamamento) pode fornecer indicações aproximadas, porém não captar integralmente o comportamento a rede de fraturas dependente do esforço poliaxial. Os resultados deste estudo possuem importantes implicações para entender o fluxo heterogêneo de fluidos geológicos (ex. águas subterrâneas e petróleo) em subsuperfície e aumentar a escala da avaliação da permeabilidade em grande escala.

## Introduction

Fractures are ubiquitous in crustal rocks in the form of faults, joints and veins, etc. These naturally occurring discontinuities often comprise complex networks and dominate hydromechanical processes in the subsurface (Zimmerman and Main 2004; Lei et al. 2017). The understanding of the nontrivial effects of natural fractures on the bulk properties of highly disordered geological media is important for many engineering applications including groundwater management, petroleum recovery, geothermal production and radioactive waste disposal (Rutqvist and Stephansson 2003).

Discrete fracture networks (DFNs) are often used to mimic naturally faulted or jointed geological systems (Dershowitz and Einstein 1988). Compared to the conventional dual porosity model (Warren and Root 1963) and analytical solution for mathematically idealised discontinuity networks (Oda 1985), the DFN approach has the advantage of explicit representation of fracture geometries together with specific description of their hydraulic transmissivities (Liu et al. 2016). The equivalent permeability tensor of a finite-sized fracture system can be derived from steady-state fluid flow simulations (Lang et al. 2014) and further used for larger-scale assessments (Blum et al. 2009). The equivalent permeability here is defined as a constant tensor in Darcy’s law to represent bulk flow in a heterogeneous medium (Renard and de Marsily 1997). It is different from the notion of effective permeability that is considered as an intrinsic material property based on the existence of representative elementary volume (REV) at a large homogenisation scale.

During the past few decades, the impact of stress on the permeability of fractured sedimentary rocks has been extensively studied based on two-dimensional (2D) fracture network models with the out-of-plane stress (perpendicular to the bedding interface) effect omitted (Zhang et al. 1996; Zhang and Sanderson 1996; Sanderson and Zhang 1999, 2004; Latham et al. 2013; Lei et al. 2014, 2015b). However, whether the effect of the out-of-plane stress is negligible may need to be examined through three-dimensional (3D) analysis. Due to the difficulties (e.g. low efficiency and meshing problem) in 3D computations, there are very few attempts to model stress-dependent fluid flow in 3D fractured rocks, an exception being a recent study by Lei et al. (2015a) which was based on an idealised 3D geometry. For more realistic fracture networks with finite-sized, non-planar discontinuities, the hydromechanical behaviour can be very different and (probably) more complex. Under in-situ stresses, finite-sized fractures aligned with the maximum principal stress direction may open and even propagate due to the high tensile stresses localised at their tips (Pollard and Segall 1987); furthermore, the sliding of pre-existing discontinuities can generate stress concentration at their ends and trigger the formation of wing/secondary cracks (Willemse and Pollard 1998). These new cracks may link pre-existing structures to form critical fluid pathways and result in an enhanced connectivity and permeability. All of these features may need to be appropriately considered in 3D simulations.

The objective of this paper is to explore the effects of polyaxial (true-triaxial) in-situ stresses on the equivalent permeability of a 3D fractured rock layer embedded with realistic joint sets. In the rest of the paper, the numerical method is briefly described, then a 3D deterministic DFN is constructed for a thin-bed limestone layer and a series of numerical experiments is designed for various polyaxial stress conditions. The simulation results about the effects of stress magnitude and orientation on the equivalent permeability of the fractured layer are presented. Finally, there is a short discussion and conclusions are drawn. This paper will mainly focus on the stress effect, whereas the complex scale effect is beyond the current scope.

## Numerical methods

### Finite-discrete element method (FEMDEM)

The numerical method used for geomechanical modelling is the combined finite-discrete element method (FEMDEM; Munjiza 2004). Extensive developments and applications of the FEMDEM method have been conducted in the past decade or so with different versions having emerged such as the code collaboratively developed by Queen Mary University of London (UK) and Los Alamos National Laboratory in the USA (Munjiza et al. 2011, 2013; Rougier et al. 2014), the Y-Geo and Irazu by the University of Toronto, Canada (Mahabadi et al. 2012; Lisjak and Grasselli 2014; Lisjak et al. 2017), and the Solidity platform by Imperial College London (Xiang et al. 2009a, b; Guo et al. 2016; Lei 2016; Lei et al. 2016). The FEMDEM model that accommodates the finite strain elasticity coupled with a smeared crack model is able to capture the complex behaviour of fractured rocks involving deformation, displacement, rotation, interaction, fracturing and fragmentation. The principles of the 3D FEMDEM model for solving stress, deformation and interaction as well as fracture propagation are similar to those of the 2D model as presented in the literature (Latham et al. 2013; Lisjak and Grasselli 2014; Lei et al. 2016). Here, only some adaptations to 3D problems are described.

**Fig. 1:**
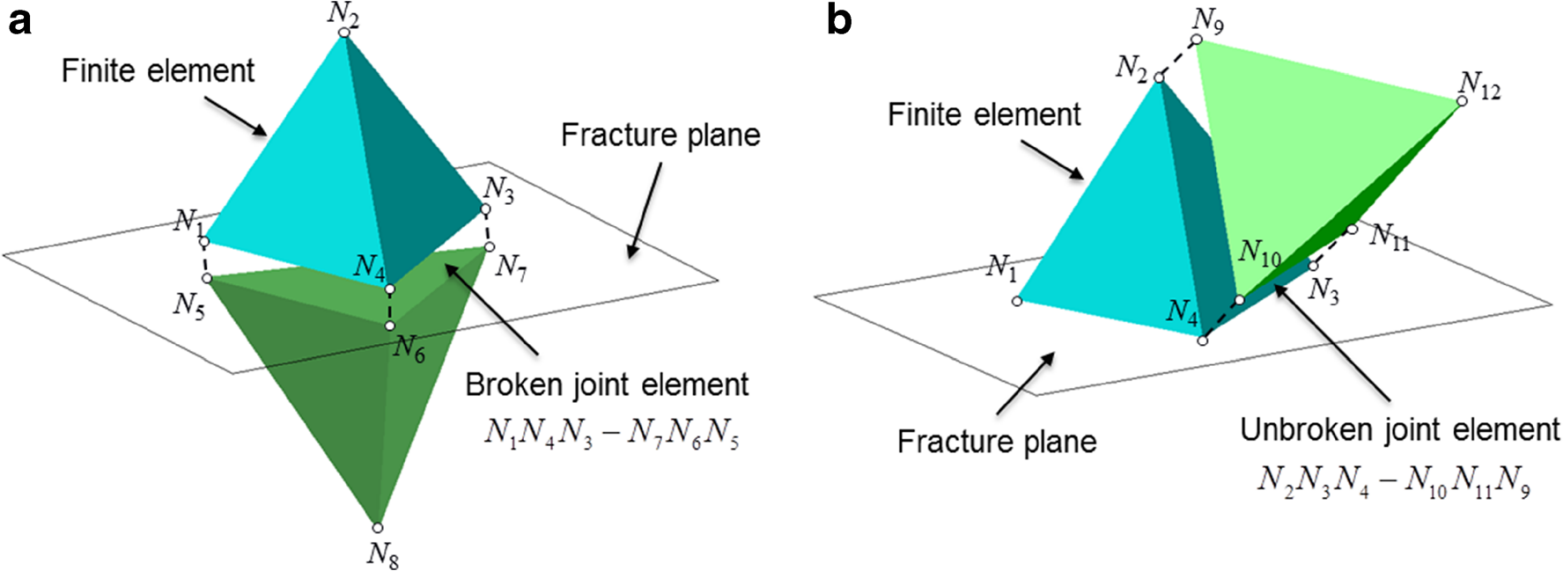
Two neighbouring tetrahedral finite elements linked by **a** a broken joint element or **b** an unbroken joint element in the 3D FEMDEM model

**Fig. 2:**
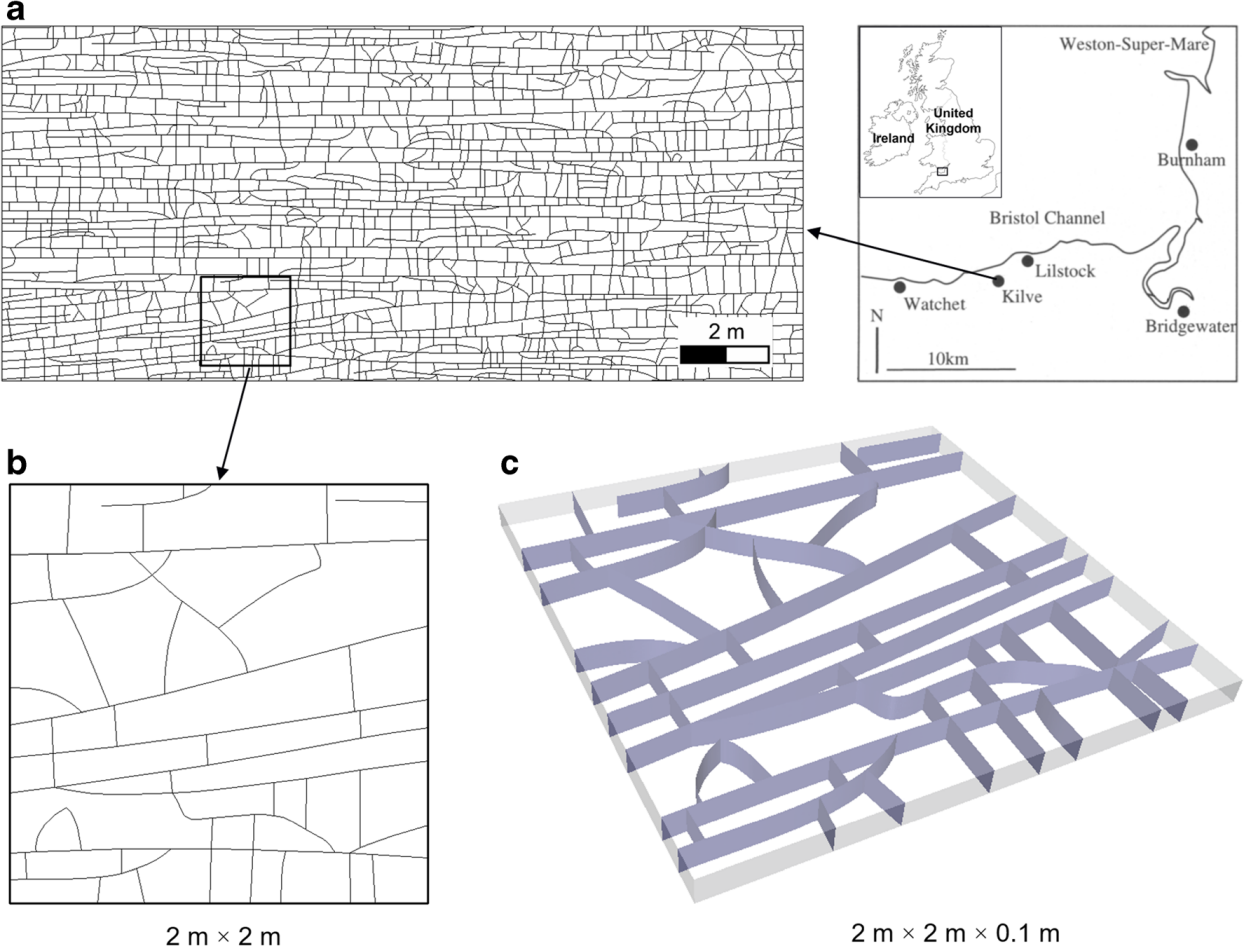
**a** An 18 m × 8 m fracture network mapped at the limestone exposure at the south margin of the Bristol Channel Basin, UK (Belayneh and Cosgrove 2004), **b** a 2 m × 2 m region is selected and **c** extruded by the layer thickness (i.e. 10 cm) to build the 3D geometry

The FEMDEM model represents a 3D fractured rock using a fully discontinuous mesh of four-noded tetrahedral finite elements and six-noded joint elements. Each tetrahedral element is connected with four joint elements and each joint element is linked to two tetrahedral volumes (Fig. 1). There are two types of joint elements (Lei et al. 2016): (1) broken joint elements which are placed along existing fractures (Fig. 1a), and (2) unbroken joint elements which are embedded inside the matrix (Fig. 1b) and may transform to broken ones as new fractures propagate under stress concentration. Both broken and unbroken joint elements are used in the FEMDEM model and inserted between the facets of tetrahedral element pairs before the simulation. No remeshing is performed in later computation.

The motions of finite elements are governed by the forces acting on elemental nodes and the governing equation is given by (Munjiza 2004):

1 $$ \mathbf{M}\ddot{\mathbf{x}}+{\mathbf{f}}_{\mathrm{int}}={\mathbf{f}}_{\mathrm{ext}} $$

where **M** is the lumped nodal mass matrix, **x** is the vector of nodal displacements, **f**_int_ are the internal nodal forces induced by the deformation of triangular elements, **f**_ext_ are the external nodal forces including external loads **f**_l_ contributed by boundary conditions and body forces, cohesive bonding forces **f**_b_ caused by the deformation of unbroken joint elements, and contact forces **f**_c_ generated by the contact interaction via broken joint elements. The solid response is modelled here in the context of Terzaghi’s effective stress law (i.e. the boundary load has eliminated the effect of pore pressure). The deformation of the bulk material is captured by linear-elastic constant-strain finite elements with the continuity constrained by the bonding forces of unbroken joint elements (Munjiza et al. 1999). The interaction of discrete matrix bodies is calculated based on the penetration of finite elements via broken joint elements (Munjiza and Andrews 2000). The elasto-plastic fracturing of geological rock media is modelled by a smeared crack (i.e. cohesive zone) method that can capture the non-linear stress-strain behaviour of the plastic zone ahead of a fracture tip (Munjiza et al. 1999). Such a crack propagation model has been recently implemented into the 3D FEMDEM formulation (Guo 2014; Guo et al. 2015, 2016). The equations of motion of the FEMDEM model are solved through an explicit time integration scheme based on the forward Euler method.

### Joint constitutive model

A joint constitutive model (JCM), similar to the one for 2D FEMDEM (Lei et al. 2016), is implemented into the 3D FEMDEM framework to simulate the deformation of rough fractures in response to normal and/or shear loading conditions. The non-linear closure of a broken joint element under compression is characterised by an empirical hyperbolic equation (Bandis et al. 1983):

2 $$ {v}_{\mathrm{n}}=\frac{\sigma_{\mathrm{n}}{v}_{\mathrm{m}}}{k_{\mathrm{n}0}{v}_{\mathrm{m}}+{\sigma}_{\mathrm{n}}} $$

where *v*_n_ is the current normal closure (mm), *σ*_n_ is the local effective normal stress (MPa) that is derived from the Cauchy stress tensor of adjacent finite elements, *k*_n0_ is the initial normal stiffness (MPa/mm), and *v*_m_ is the maximum allowable closure (mm). Values of *k*_n0_ and *v*_m_ are given by (Bandis et al. 1983):

3 $$ {k}_{\mathrm{n}0}=-7.15+1.75\ \mathrm{JRC}+0.02\times \frac{\mathrm{JCS}}{a_0} $$

4 $$ {v}_{\mathrm{m}}=-0.1032-0.0074\ \mathrm{JRC}+1.1350\times {\left(\frac{\mathrm{JCS}}{a_0}\right)}^{-0.2510} $$

where *a*_0_ is the initial aperture (mm), JRC is the joint roughness coefficient, and JCS is the joint compressive strength (MPa). Coefficients derived from experimental measurements of numerous joint samples of five different rock types under a third loading cycle are adopted since in-situ fractures are considered more likely to behave in a manner similar to the third or fourth cycle (Barton et al. 1985). These empirical equations and coefficients can statistically interpret the observed behaviour of the experimental samples under the specific testing conditions (Bandis et al. 1983). However, attention may be needed if they are applied to actual engineering problems (Baghbanan and Jing 2008). Both JRC and JCS are scale-dependent parameters (Bandis et al. 1981) and their field-scale values, i.e. JRC_n_ and JCS_n_, can be estimated using (Barton et al. 1985):

5 $$ {\mathrm{JRC}}_{\mathrm{n}}={\mathrm{JRC}}_0{\left(\frac{L_{\mathrm{n}}}{L_0}\right)}^{-0.02\ {\mathrm{JRC}}_0} $$

6 $$ {\mathrm{JCS}}_{\mathrm{n}}={\mathrm{JCS}}_0{\left(\frac{L_{\mathrm{n}}}{L_0}\right)}^{-0.03\ {\mathrm{JRC}}_0} $$

where *L*_n_ is the field-scale effective joint length (i.e. size of a block edge between fracture intersections) defined by the spacing of cross-joints, JRC_0_ and JCS_0_ are measured based on the laboratory sample with length *L*_0_.

During the shearing process under a normal compression, fractures contract first due to the compressibility of asperities and then dilate with roughness damaged and destroyed (Barton et al. 1985). Dilational displacement can be related to the shear displacement using an incremental formulation given by (Olsson and Barton 2001):

7 $$ \mathrm{d}{v}_{\mathrm{s}}=- \tan {d}_{\mathrm{mob}}\mathrm{d} u $$

where d*v*_s_ is the increment of normal displacement caused by shear dilation, d*u* is the increment of shear displacement, and *d*_mob_ is the mobilised tangential dilation angle given by (Olsson and Barton 2001):

8 $$ {d}_{\mathrm{mob}}=\frac{1}{M}{\mathrm{JRC}}_{\mathrm{mob}}{ \log}_{10}\left(\frac{{\mathrm{JCS}}_{\mathrm{n}}}{\sigma_{\mathrm{n}}}\right) $$

where *M* is a damage coefficient given by (Barton and Choubey 1977):

9 $$ M=\frac{{\mathrm{JRC}}_{\mathrm{n}}}{12\ { \log}_{10}\left(\frac{{\mathrm{JCS}}_{\mathrm{n}}}{\sigma_{\mathrm{n}}}\right)}+0.70 $$

The mobilised joint roughness coefficient JRC_mob_ can be calculated using a dimensionless model (Barton et al. 1985) based on the ratio of the current shear displacement to the peak shear displacement *u*_p_, which is given by (Barton et al. 1985):

10 $$ {u}_{\mathrm{p}}=\frac{L_{\mathrm{n}}}{500}{\left(\frac{{\mathrm{JRC}}_{\mathrm{n}}}{L_{\mathrm{n}}}\right)}^{0.33} $$

The coupled normal and shear deformation can thus be modelled by an incremental formulation:

11 $$ \mathrm{d} v=\mathrm{d}{v}_{\mathrm{n}}+\mathrm{d}{v}_{\mathrm{s}} $$

The mechanical aperture *a*_m_ is derived by combing the effects of mesoscopic opening (induced by fracture network deformation and explicitly resolved in the FEMDEM) and microscopic closure (controlled by fracture roughness and implicitly captured by the JCM) as given by (Lei et al. 2015a, 2016):

12 $$ {a}_{\mathrm{m}}=\left\{\begin{array}{cc}\hfill {a}_0+ w\hfill & \hfill, w\ge 0\hfill \\ {}\hfill {a}_0- v\hfill & \hfill, w<0\hfill \end{array}\right. $$

where *w* is the mesoscopic normal separation of the opposite walls of broken joint elements in the deformed FEMDEM mesh, and *v* is the microscopic accumulative closure derived from the JCM incremental calculation. The first part of the piecewise function corresponds to the scenario that the broken joint element is mesoscopically opened, while the second part models the condition that the two opposite walls of the fracture are in contact at the FEMDEM grid scale. The hydraulic aperture *a*_h_ defined as an equivalent aperture for laminar flow is derived based on an empirical relation with the mechanical aperture (Olsson and Barton 2001):

13 $$ {a}_{\mathrm{h}}=\left\{\begin{array}{cc}\hfill {a}_{\mathrm{m}}^2/{\mathrm{JRC}}^{2.5}\hfill & \hfill, \kern0.5em  u/{u}_{\mathrm{p}}\le 0.75\hfill \\ {}\hfill {a}_{\mathrm{m}}^{1/2}{\mathrm{JRC}}_{\mathrm{m}\mathrm{ob}}\hfill & \hfill, \kern0.5em  u/{u}_{\mathrm{p}}\ge 1.0\kern0.5em \hfill \end{array}\right. $$

A linear interpolation is used to determine the hydraulic aperture in the transition phase, i.e. 0.75 < *u*/*u*_p_ < 1.0.

### Fracture and matrix flow modelling

Fluid flow through the fractured rock with multiple intersecting fractures and permeable matrix is solved using the combined finite element-finite volume method (Geiger et al. 2004). Single-phase steady state flow of incompressible fluid with constant viscosity through porous media, in absence of sources and sinks, is governed by the continuity equation and Darcy’s law, which are reduced to a Laplace equation as:

14 $$ \nabla \cdot \left( k\nabla p\right)=0 $$

where *k* is the intrinsic and isotropic permeability of the porous media with local variability permitted, and *p* is the fluid pressure solved at the nodes of unstructured finite element grids by employing the standard Galerkin method. The element-wise constant barycentric velocity is resolved based on the pressure gradient vector field by applying Darcy’s law given by:

15 $$ {\mathbf{u}}^{\mathbf{e}}=-\frac{k^{\mathrm{e}}}{\mu}\nabla {p}^{\mathrm{e}} $$

where **u** ^**e**^ is the vector field of element-wise constant velocities, *p* ^e^ is the local element pressure field, *μ* is the constant fluid viscosity, and *k* ^e^ is the local permeability of a matrix volumetric element with an assumed constant value or a lower dimensional fracture element having a variable value related to the local hydraulic aperture *a*_h_ obeying the cubic law for laminar flow between parallel plates (Witherspoon et al. 1980). By applying a prescribed macroscopic pressure differential on each pair of opposite boundary surfaces with no-flow conditions on the remaining ones parallel to the flow direction, pressure diffusion is solved for all fracture and matrix elements of the entire domain. The equivalent permeability of the fractured media is computed using element volume weighted averaging of pressure gradients and fluxes for elements *e* within a restricted subvolume *V* of the flow region away from the borders to eliminate boundary effects (Lang et al. 2014):

16 $$ \frac{1}{V}\sum_{\mathrm{e}}\underset{V^{\mathrm{e}}}{\int }{u}_j^{\mathrm{e}}{dV}^{\mathrm{e}}=\frac{k_{i j}}{\mu}\frac{1}{V}\sum_{\mathrm{e}}\underset{V^{\mathrm{e}}}{\int}\frac{\partial {p}^{\mathrm{e}}}{\partial {x}_i}{dV}^{\mathrm{e}} $$

where *u* ^e^_*j*_ is the element-wise barycentric velocity in the *j* direction, *∂p* ^e^/*∂x*_*i*_ is the element pressure gradient along *x*_*i*_, and *k*_*ij*_ is the components of the symmetric second-rank permeability tensor **k**:

17 $$ \mathbf{k}=\left[\begin{array}{ccc}\hfill {k}_{xx}\hfill & \hfill {k}_{xy}\hfill & \hfill {k}_{xz}\hfill \\ {}\hfill {k}_{yx}\hfill & \hfill {k}_{yy}\hfill & \hfill {k}_{yz}\hfill \\ {}\hfill {k}_{zx}\hfill & \hfill {k}_{zy}\hfill & \hfill {k}_{zz}\hfill \end{array}\right] $$

For more details about solving the fracture and matrix flow, the reader is referred to the work by Geiger et al. (2004) and Lang et al. (2014).

## Model setup

The fracture network used in this research is based on the outcrop map of a limestone bed located at Kilve on the southern margin of the Bristol Channel Basin, UK (Fig. 2a; Belayneh and Cosgrove 2004). This fractured limestone (10 cm thick) is sandwiched between almost impervious shales and the vertically dipping joints are layer bound (not extending into the neighbouring shales). The joint network exhibits a ladder pattern consisting of two major sets. The E–W striking set (set 1) that formed in an early stage contains “through-going” (or persistent) fractures. The N–S striking set (set 2) that developed later consists of short joints abutting the fractures of set 1. It can be noted that this highly hierarchical joint pattern is featured by “T” (i.e. abutting) and “X” (i.e. crossing) type nodes with only a few “I” type nodes (i.e. terminating inside matrix). Considering the very expensive runtime of 3D FEMDEM calculation, a 2 m × 2 m region (Fig. 2b) is selected from the original 18 m × 8 m analogue for geomechanical modelling (the selected domain requires a run-time of ~220 h on a desktop computer equipped with an Intel(R) Xeon(R) CPU E5–2697@2.30 GHz). The extracted 2D network is extruded by 10 cm (i.e. the thickness of the layer) to build a 3D geometry (Fig. 2c). Material properties are assumed to represent typical fractured limestone (Lama and Vutukuri 1978; Bandis et al. 1983) as given in Table 1.

**Table 1:** Material properties of the fractured limestone

Property	Value
Bulk density *ρ* (kg/m^3^)	2,700
Young’s modulus *E* (GPa)	30
Poisson’s ratio *υ*	0.27
Tensile strength *f*_t._ (MPa)	7
Internal friction angle *ϕ*_i_ (°)	26.6
Cohesion *c* (MPa)	15
Mode I energy release rate *G*_I_ (J/m^2^)	100
Mode II energy release rate *G*_II_ (J/m^2^)	400
Matrix permeability *k*_m_ (m^2^)	1 × 10^−15^
Residual friction angle *ϕ*_r_ (°)	31
Laboratory sample length *L*_0_ (m)	0.2
JCS_0_ (MPa)	169
JRC_0_	9.7
Initial mechanical aperture *a*_0_ (mm)	0.194

**Fig. 3:**
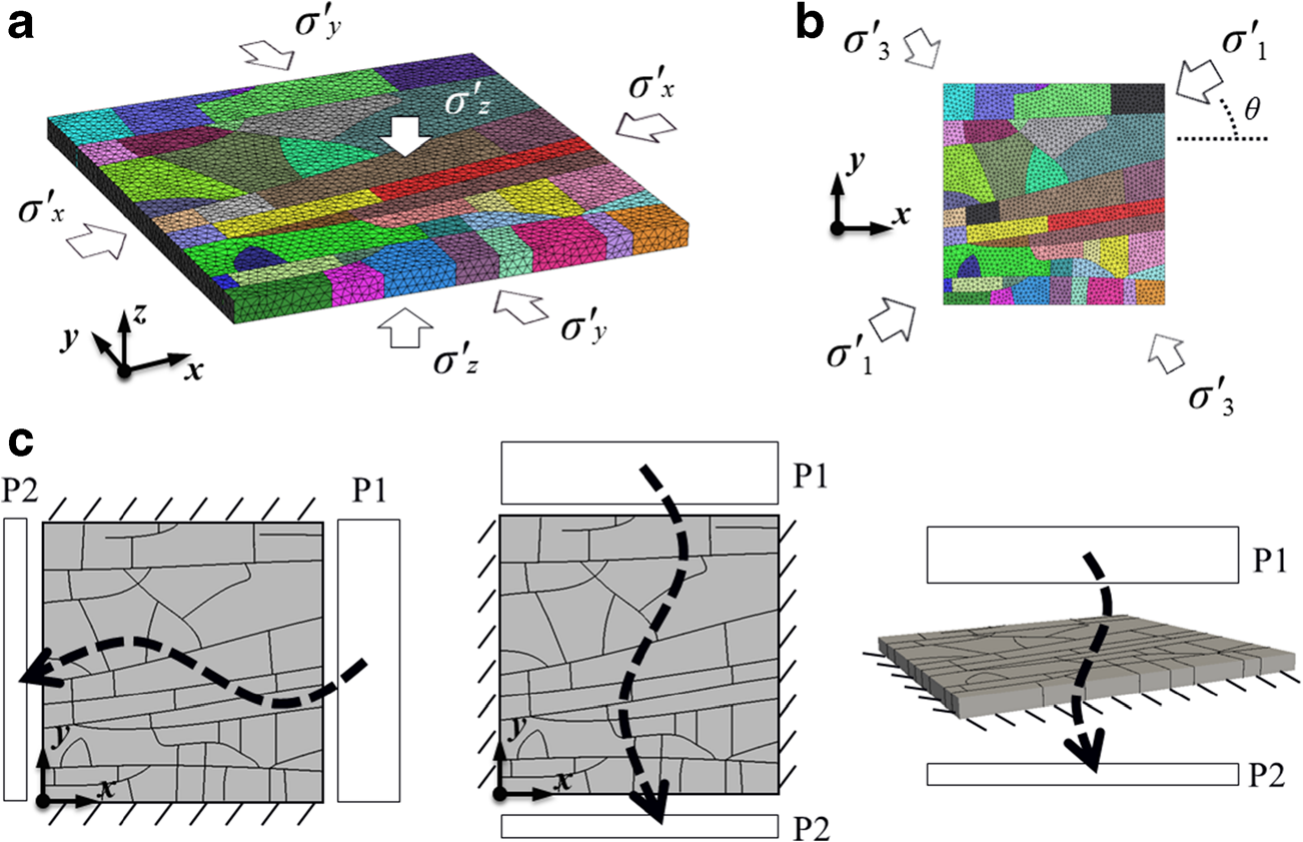
Procedure for the numerical experiment: geomechanical modelling with polyaxial effective stresses applied **a** orthogonally and **b** obliquely to the problem domain, and **c** calculation of the equivalent permeability based on single-phase steady-state fluid flow through the stressed layer under a prescribed macroscopic pressure differential imposed on each pair of opposite boundaries, while the remaining ones are impervious (*σ′*_*x*_, *σ′*_*y*_ and *σ′*_*z*_ denote the far-field stress components in the x, y and z directions, respectively; *σ′*_*1*_ and *σ′*_*3*_ denote the maximum and minimum far-field principal stresses, respectively; *θ* indicates the angle of rotation of the far-field stress field with respect to the z axis; *P1–P2* gives the fluid pressure drop across the rock)

The problem domain containing pre-existing fractures is discretised by an unstructured mesh with an average element size of ~3.0 cm (97,606 tetrahedral finite elements and 201,502 triangular joint elements in total). The geomechanical behaviour of the fractured layer in response to polyaxial effective in-situ stresses is explored with respect to the variation of stress magnitude and orientation. For the study of the stress magnitude effect, the far-field stresses are loaded orthogonally to the model domain (Fig. 3a), with *σ′*_x_, *σ′*_y_ and *σ′*_z_ varying between 5 and 15 MPa. For analysing the orientation effect, the polyaxial far-field stress field (*σ′*_1_ = 15 MPa, *σ′*_3_ = 5 MPa and *σ′*_z_ = 5 ~ 15 MPa) is applied at different angles (*θ* = 30°, 60°, 90°, 120° and 150°) to the rock (Fig. 3b). The gravitational body forces are neglected for this thin layer. The role of pore fluid pressure is assumed to be a second-order factor for aperture development and the Biot-type poroelastic effect is only considered for a particular scenario with the Biot coefficient equal to 1.0. Single-phase steady-state fluid flow through the deformed fracture network with stress-induced variable apertures is further modelled by imposing the classical permeameter boundary condition: two opposite boundary surfaces of the rectangular volume domain have a fixed pressure drop (i.e. 1 kPa), while the four orthogonal boundaries parallel to the flow direction are impervious (Fig. 3c). Matrix permeability *k*_m_ is assumed to be 1 × 10^−15^ m^2^, which gives a high fracture-matrix permeability contrast so that the flow is dominated by fractures (Matthäi and Belayneh 2004). To analyse the distribution of vertical flow under the prescribed vertical pressure drop, a 20 × 20 square grid is superimposed over the finite element system. The flow rate of each square area is computed by summing the fluxes of the finite element nodes inside the local square. This approach provides a way to characterise and visualise the heterogeneous distribution of vertical flow through the fractured rock layer (Sanderson and Zhang 1999).

## Results

### Effect of stress magnitude

**Fig. 4:**
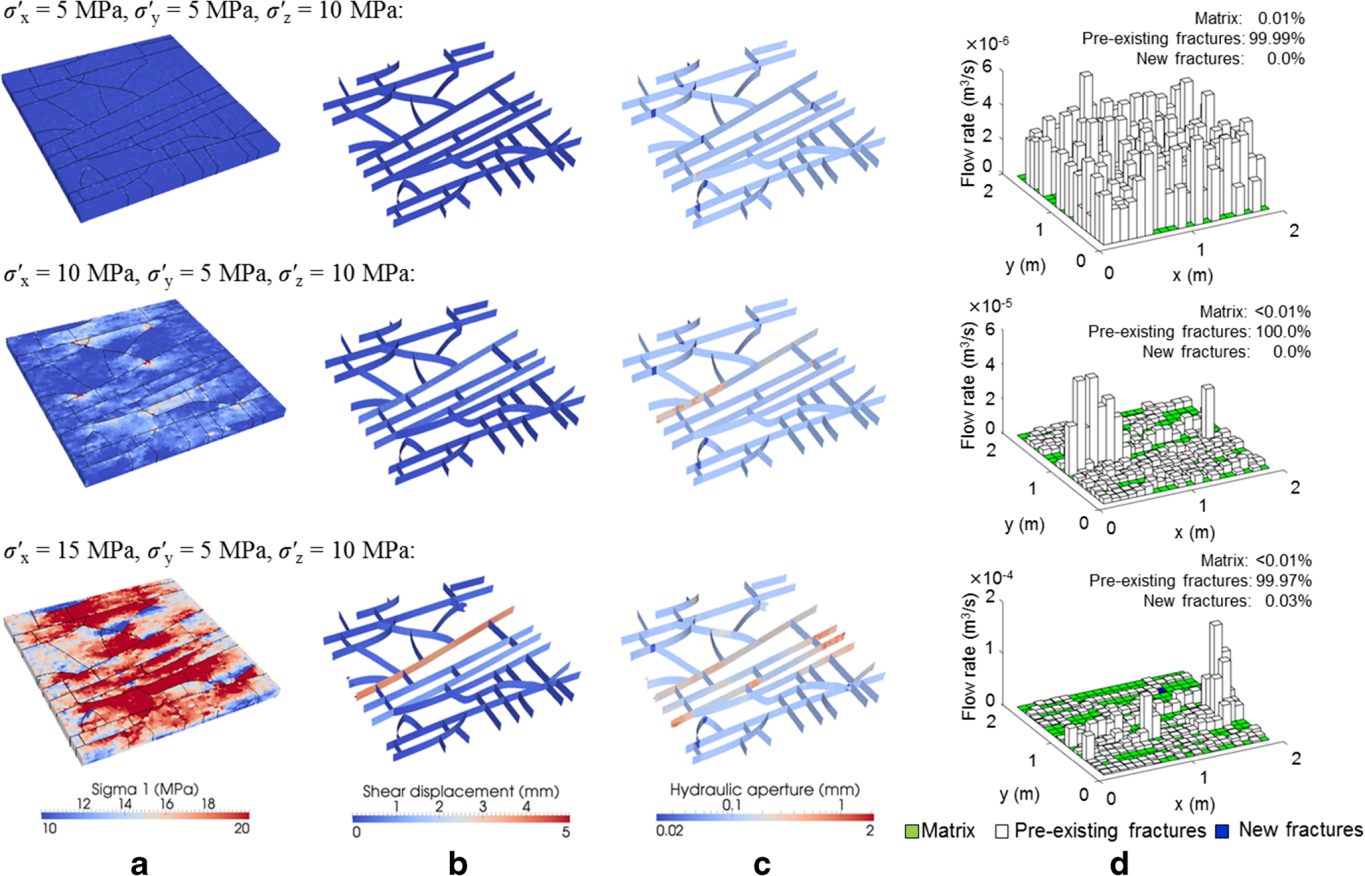
Distributions of **a** local maximum principal stresses, **b** shear displacements, **c** hydraulic apertures and **d** vertical flow rates (under a prescribed vertical pressure drop of 1 kPa) in the fractured layer under polyaxial stress conditions with a varied *σ′*_*x*_ = 5, 10, or 15 MPa and fixed *σ′*_*y*_ = 5 MPa, *σ′*_*z*_ = 10 MPa. Note that in **d**, cubes with different colours, i.e. *green*, *white* and *blue*, correspond to the flow through matrix, pre-existing fractures and new fractures, respectively, and the flow rate axis scales differently in different cases

Figure 4 presents the simulation results with different *σ′*_x_ ranging from 5 to 15 MPa, but the same *σ′*_y_ = 5 MPa and *σ′*_z_ = 10 MPa. With the increase of *σ′*_x_, the distribution of local maximum principal stresses in the rock changed from a homogeneous pattern to a highly heterogeneous field with the high stress zones aligning the direction of *σ′*_x_ (Fig. 4a). The increased horizontal stress ratio of *σ′*_x_/*σ′*_y_ also triggered the sliding of pre-existing fractures (especially the persistent ones; Fig. 4b), which accommodated large apertures, caused slight crack propagation (Fig. 4c) and formed highly localised channels for vertical flow (Fig. 4d). The presence of flow in the squares with no fractures (see green cubes in Fig. 4d) is attributed to the nonzero matrix permeability. As shown in Fig. 4d, fluid is predominantly transported through the zones with pre-existing fractures and flow localisation under the increased horizontal stress ratio tends to be mainly induced by the shear dilation of pre-existing fractures.

**Fig. 5:**
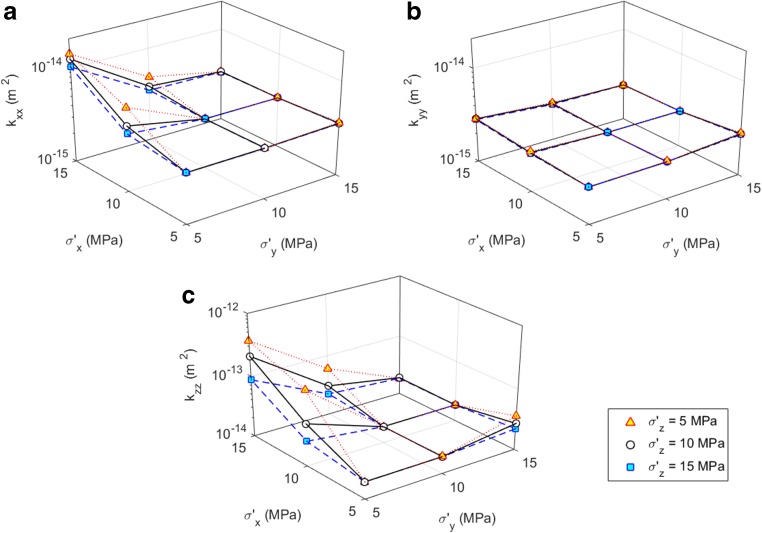
Variation of the equivalent permeability components **a** *k*_xx_, **b** *k*_yy_, and **c** *k*_zz_ of the fractured rock layer under various polyaxial stress conditions (*σ′*_x_, *σ′*_y_, *σ′*_z_ = 5, 10 or 15 MPa, i.e. 27 stress scenarios)

Simulations were conducted for all combinations of *σ′*_x_, *σ′*_y_, *σ′*_z_ = 5, 10 or 15 MPa (i.e. 27 stress scenarios) and the stress-dependent equivalent permeability is presented in Fig. 5. The bed-normal permeability, i.e. *k*_zz_, of the fractured layer is about one order of magnitude larger than the horizontal components, i.e. *k*_xx_ and *k*_yy_. Note that *k*_xx_ is larger than *k*_yy_ due to the better connectivity of the joint pattern in the x direction, i.e. the strike direction of the dominant persistent fractures. It can be noticed that the variation of the equivalent permeability exhibits distinct behaviour in the x and y directions. The equivalent permeability is much more sensitive to an increased *σ′*_x_/*σ′*_y_ than to an increased *σ′*_y_/*σ′*_x_, due to the geometrical anisotropy of the joint network. *k*_*zz*_ also seems to be more sensitive to the stress variation than *k*_xx_ and *k*_yy_. It can be seen that the permeability contrast of *k*_zz_/*k*_yy_ spans over almost two orders of magnitude when *σ′*_x_/*σ′*_y_ = 3; furthermore, an increased vertical stress *σ′*_z_ seems to reduce the equivalent permeability, which may be attributed to the more closed apertures under higher mean stresses. The results demonstrate that the magnitude and ratio of the far-field stresses have important impact on the permeability of the fractured layer.

### Effect of stress orientation

**Fig. 6:**
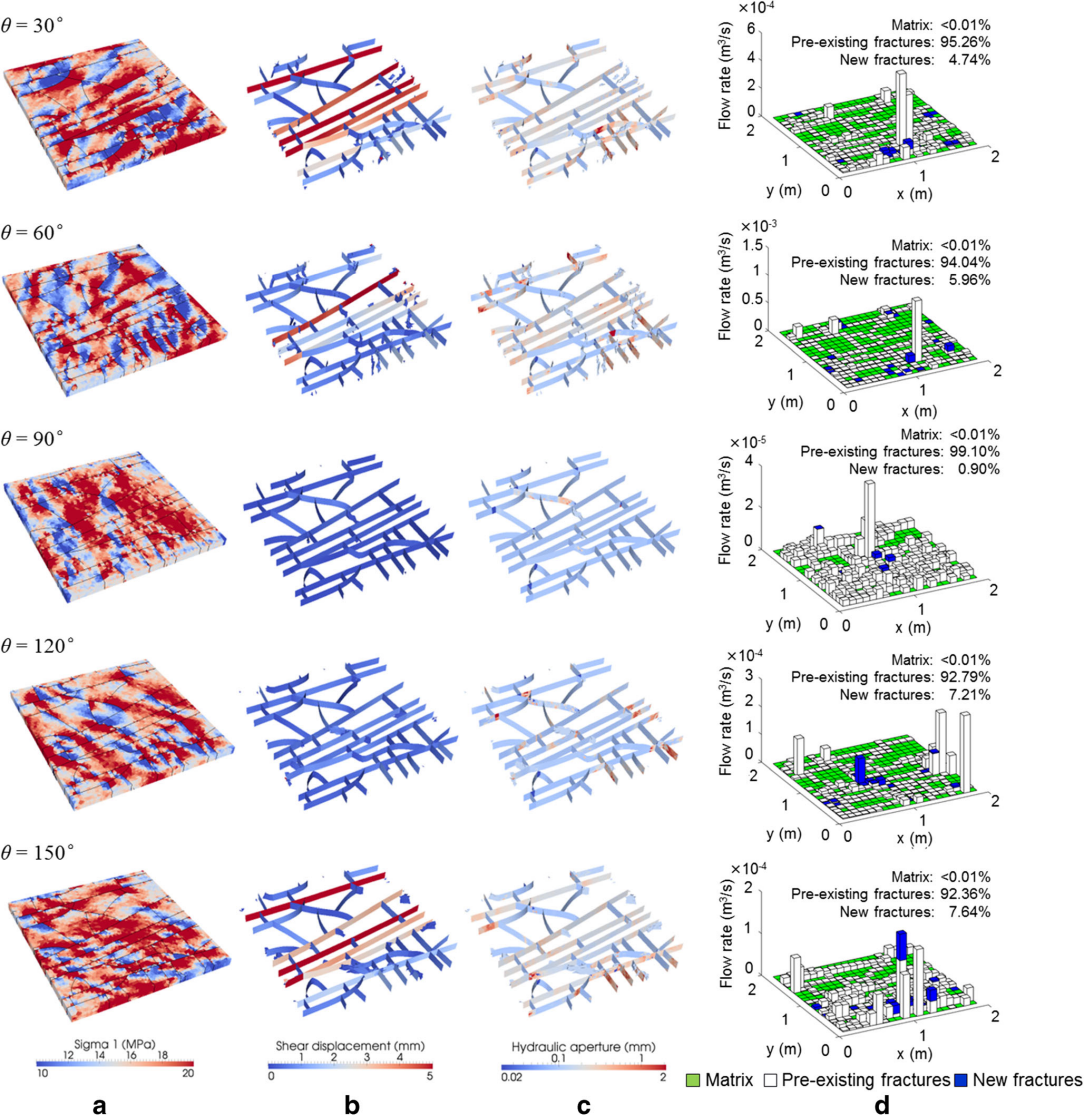
Distributions of **a** local maximum principal stresses, **b** shear displacements, **c** hydraulic apertures and **d** vertical flow rates (under a prescribed vertical pressure gradient of 1 kPa) in the fractured layer under the polyaxial stresses of *σ′*_1_ = 15 MPa, *σ′*_3_ = 5 MPa, *σ′*_z_ = 10 MPa applied at different angles. Note that in **d**, cubes with different colours, i.e. *green*, *white* and *blue*, correspond to the flow through matrix, pre-existing fractures and new fractures, respectively, and the flow rate axis scales differently in different cases

**Fig. 7:**
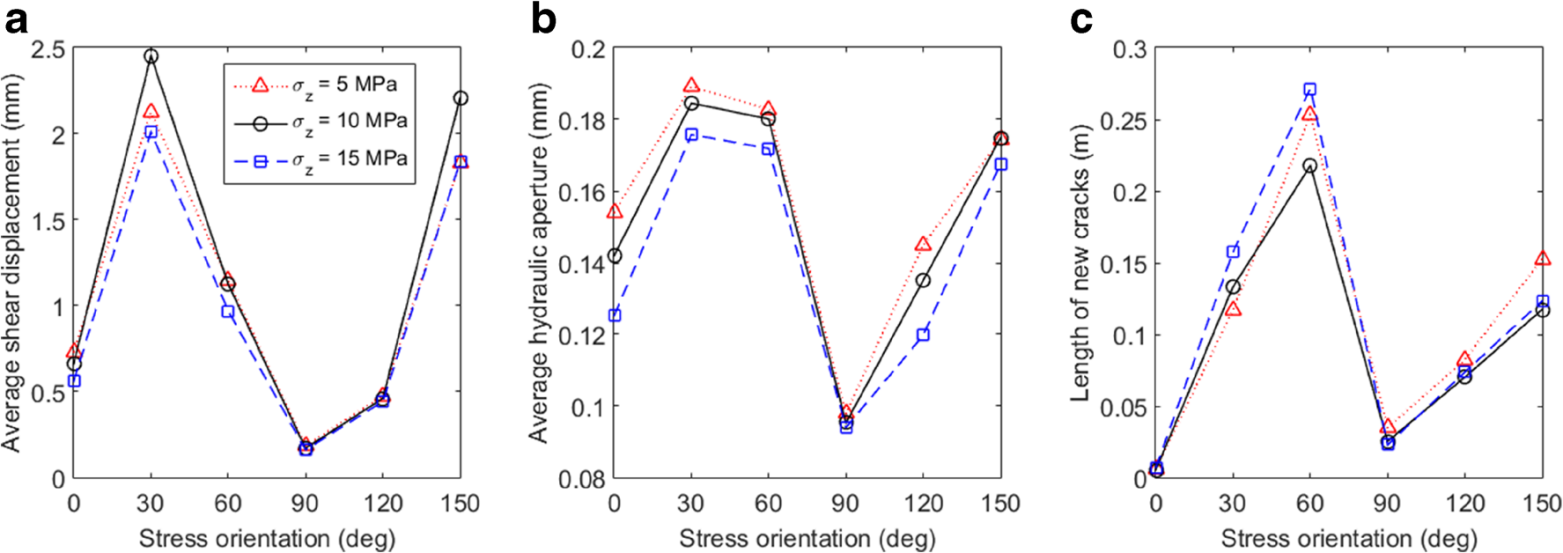
Variation of **a** the average shear displacement, **b** average hydraulic aperture, and **c** length of new cracks with the orientation change of the polyaxial far-field stress fields with *σ′*_*1*_ = 15 MPa, *σ′*_*3*_ = 5 MPa, *σ′*_*z*_ = 5, 10 or 15 MPa

**Fig. 8:**
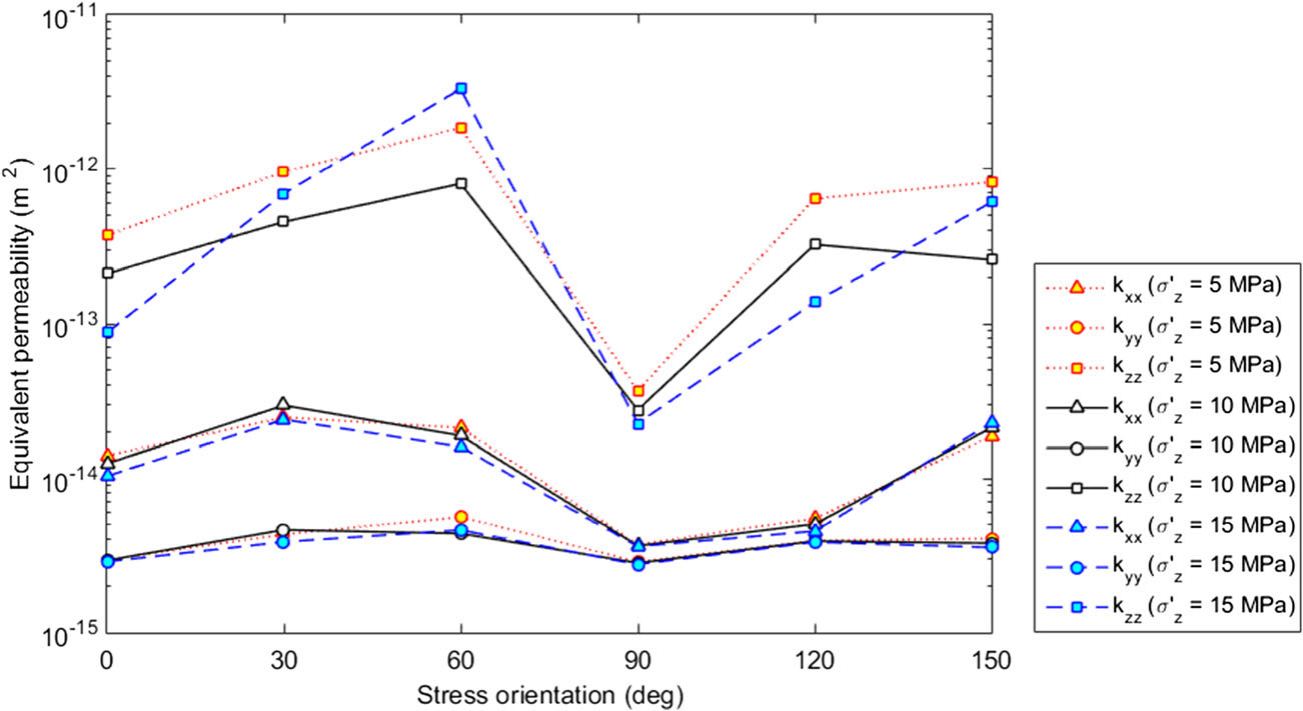
Variation of the equivalent permeability of the fracture layer with the orientation change of the polyaxial far-field stress fields with *σ′*_*1*_ = 15 MPa, *σ′*_*3*_ = 5 MPa, *σ′*_*z*_ = 5, 10 or 15 MPa

Figure 6 compares the simulation results of rotating a polyaxial far-field stress field (*σ′*_1_ = 15 MPa, *σ′*_3_ = 5 MPa and *σ′*_z_ = 10 MPa) by a range of angles *θ* with the z direction as the axis of rotation. Note *θ* = 0° corresponds the case of *σ′*_x_ = 15 MPa, *σ′*_y_ = 5 MPa and *σ′*_z_ = 10 MPa as has been shown in Fig. 4. The spatial distribution of local maximum principal stresses varies according to the stress field rotation, with the high stress bands formed along the direction of *σ′*_1_ (Fig. 6a). Compared to the cases with orthogonally loaded stresses (i.e. *θ* = 0° and 90°), the fracture sets under oblique stress fields are more preferentially oriented for shearing with wider hydraulic apertures (caused by shear dilation and block rotation), more new cracks and stronger flow localisation generated (Fig. 6b,c,d). This is further confirmed by Fig. 7 (see the solid lines), which shows the variation of the average shear displacement, average hydraulic aperture and length of new cracks of the fracture network in response to the change of the stress orientation. The fracture network in the 30° and 150° cases experienced the most intensive shearing; fractures in the 30°, 60° and 150° cases are associated with the larger hydraulic apertures; new cracks propagated most in the 60° case; thus, the fractured layer exhibited much higher permeability under obliquely loaded far-field stresses (Fig. 8). As shown in Fig. 6d, the flow localisation is also dominated by the shear dilation of pre-existing fractures (contributing to >90% of the total flow), while the propagation of new fractures has minor impacts (contributing to <10% of the total flow).

The vertical stress *σ′*_z_ seems to have some important influence on fracture and flow behaviour. The polyaxial stress condition with *σ′*_z_ = 10 MPa tends to trigger more sliding on fracture walls (Fig. 7a), which may be explained by the relation of *σ′*_3_ < *σ′*_z_ < *σ′*_1_ that concentrates differential stresses to develop horizontally, whereas in other cases *σ′*_z_ is either equal to *σ′*_1_ or *σ′*_3_. An increased *σ′*_z_ tends to enhance the compression of fracture walls (due to increased mean stresses) and therefore reduces fracture apertures (Fig. 7b). Furthermore, more new cracks emerged in the cases of *σ′*_z_ = 5 and 15 MPa (Fig. 7c), because such stress conditions tend to assist the propagation either horizontally along the direction of *σ′*_1_ (when *σ′*_z_ = 5 MPa) or vertically through the layer thickness (when *σ′*_z_ = 15 MPa). The role of the overburden stress in fracture shearing, aperture change and crack propagation resulted in a complex phenomenon that the increased *σ′*_z_ can lead to either increased or decreased permeability in different orientation scenarios (Fig. 8). It is noted that *σ′*_z_ has negligible influence on *k*_xx_ and *k*_yy_, but can significantly affect *k*_zz_ (although the variation is within one order of magnitude).

## Discussion

Stress-controlled variability of fracture displacements and apertures in a 3D fractured sedimentary layer embedded with realistic joint sets has been simulated using the 3D FEMDEM model. The behaviour of fractures such as shearing, opening and propagating is significantly influenced by the magnitude and orientation of the polyaxial stress field. When pre-existing fractures are preferentially oriented to the far-field stresses associated with a high stress ratio, considerable shearing would occur and further trigger the rotation of matrix blocks, with some very large openings created (along block boundaries) and strongly heterogeneous fluid flows induced. The simulation results show consistency with previous field observations: only a small portion of fractures are highly conductive (Tsang and Neretnieks 1998; Follin et al. 2014); critically stressed faults tend to have much higher hydraulic conductivity (Barton et al. 1995; Zoback 2007). The observation that the shear dilation of pre-existing fractures has dominant effects on flow localisation based on our simulations is also consistent with the results of previous numerical studies (Min et al. 2004; Baghbanan and Jing 2008; Zhao et al. 2011). Compared to the results based on an idealised 3D persistent fracture network (Lei et al. 2015a), the permeability of this fractured layer is less sensitive to stress changes, because the matrix blocks of this limestone layer are partially bounded by impersistent fractures and tend to be more difficult to rotate under more restrictive interlocking between blocks. Furthermore, the important role of the out-of-plane stress (perpendicular to the bedding interface) inferred from the simulation results (Figs. 5, 7 and 8) suggests that 2D models (Zhang et al. 1996; Zhang and Sanderson 1996; Sanderson and Zhang 1999, 2004; Latham et al. 2013; Lei et al. 2014) may provide indicative approximations but not fully capture the polyaxial stress-dependent behaviour of 3D fractured layers, which requires 3D modelling.

Despite the great capability of the 3D model developed, some limitations may still exist—for example, the fracture behaviour was modelled based on an empirical formulation that assumes isotropic roughness properties; however, both laboratory and numerical experiments have shown that fracture apertures evolve anisotropically under shearing and form more pronounced channels perpendicular to the shear direction (Yeo et al. 1998; Koyama et al. 2006). Such an anisotropic effect may lead to even higher bed-normal permeability and more localised vertical flow in the fractured layer. To simulate it, a 3D anisotropic joint constitutive model, e.g. the one proposed by Jing et al. (1994), would need to be implemented into the 3D FEMDEM framework. Another limitation is that initial apertures were assumed constant for the whole joint network. The important correlation between fracture apertures and fracture sizes may be modelled using a linear (Pollard and Segall 1987) or sublinear (Olson 2003) correlation, while the intrinsic heterogeneity of fracture wall asperities may be further modelled by fractal (Thompson and Brown 1991) or self-affine (Oron and Berkowitz 1998) profiles. The complex relation between mechanical and hydraulic apertures of rough fractures can also have important influences on the fluid flow behaviour (Luo et al. 2016); furthermore, due to the very expensive run-time of the 3D FEMDEM calculation based on the current processing power, the modelling domain is limited to metric scale. To compute larger-scale problems, parallelisation techniques may be employed in the FEMDEM computation (Lukas et al. 2014). In addition, the method of upscaling small-scale modelling results with statistical and geomechanical properties preserved (Lei et al. 2015b) can also be a possible solution. Extensions of this 3D work also include hydromechanical modelling of a fractured multilayer system, such as the one constructed in Wang et al. (2017), where the fluid flow in bedding planes is also stress-dependent and can influence the vertical flow through the layers. One more avenue for future work is the analysis of more general 3D fracture networks with randomly oriented fractures, which are more indicative of crystalline rocks; however, the difficulty of meshing such complex geometries involving very small intersection angles needs to be tackled.

## Conclusions

To conclude, this paper presented a study of the stress, deformation and fluid flow in a 3D fractured rock layer embedded with a realistic joint network under various polyaxial stress conditions. Geomechanical behaviour of the fractured layer was simulated by the 3D FEMDEM model combined with a joint constitutive model and a crack propagation model. Important rock and fracture responses have been captured including the normal/shear deformation of pre-existing fractures and the propagation of new fractures. The polyaxial stress field with a high stress ratio and/or an oblique orientation to fracture sets can result in strongly heterogeneous distributions of stresses, shear displacements and fracture apertures. The fractured layer also tends to exhibit stronger flow localisation and higher equivalent permeability as the far-field stress ratio is increased and the stress field is rotated such that fractures are preferentially oriented for shearing. The shear dilation of pre-existing fractures has dominant effects on flow localisation in the system, while the propagation of new fractures has minor impacts. The role of the overburden stress suggests that the conventional 2D analysis that neglects the out-of-plane stress effect may provide indicative approximations but not fully capture the polyaxial stress-dependent behaviour of 3D fractured layers. The results of this study have important implications for upscaling permeability to grid block properties for reservoir flow simulation and exploring mineral deposits for the mining industry.
